# Photobiomodulation and the oral-gut microbiome axis: therapeutic potential and challenges

**DOI:** 10.3389/fmed.2025.1555704

**Published:** 2025-04-09

**Authors:** Neda Hakimiha, Somayeh Jahani Sherafat, E-Liisa Laakso, Reza Fekrazad

**Affiliations:** ^1^Laser Application in Medical Sciences Research Center, Shahid Beheshti University of Medical Sciences, Tehran, Iran; ^2^Mater Research Institute, University of Queensland, South Brisbane, QLD, Australia; ^3^School of Health Sciences and Social Work, Griffith University, Brisbane, QLD, Australia; ^4^Radiation Sciences Research Center (RSRC), AJA University of Medical Sciences, Tehran, Iran; ^5^International Network for Photo Medicine and Photo Dynamic Therapy (INPMPDT), Universal Scientific Education and Research Network (USERN), Tehran, Iran

**Keywords:** microbiome, gastrointestinal, low-level light therapy, oral-gut axis, oral cavity, photobiomodulation

## Abstract

This Perspective article explores the challenges associated with the direct application of photobiomodulation (PBM) to the gut and presents a novel hypothesis for indirect gut health modulation through oral microbiome alteration. Given the difficulties in delivering PBM effectively to deep gastrointestinal tissues, an alternative approach involves targeting the oral microbiome, which has a demonstrated relationship with the gut microbiome. Research indicates that PBM applied to the oral cavity could selectively alter microbial composition. This alteration may, via the oral-gut microbiome axis, indirectly impact gut health. This hypothesis, supported by preliminary studies, suggests that oral PBM could offer a promising non-invasive strategy for managing gut-related disorders. Furthermore, there may be a link between the oral microbiome and brain diseases. Given the proximity to the brain, PBM-induced changes in the oral microbiota could indirectly help prevent neurological disorders. However, further investigation is necessary to comprehensively elucidate the underlying mechanisms and therapeutic implications of this approach.

## 1 Introduction

Numerous systemic diseases, particularly those associated with chronic inflammation, have recently been linked to the state of the gut microbiome (1). While the precise characteristics of a “healthy” gut microbiome remain undefined, growing evidence has identified specific microbial phyla and genera that promote gut health, as well as those that may contribute to disease (2). Research is increasingly focused on strategies to shift the microbiome toward a healthier state, including advancements in the emerging field of “photobiomics” (3, 4). The concept of “photobiomics” pertains to the examination of the effects of light on metabolic processes and the microbiome, along with the interactions that occur between these two components. Additionally, the relationship between the oral and gut microbiomes, known as the oral-gut microbiome axis, offer a novel avenue for further research (4).

This article aimed to examine the intricate relationship between the oral and gut microbiomes, addresses the difficulties associated with directly applying light to the gut, and introduces a novel hypothesis suggesting that gut health can be indirectly affected by changes in the oral microbiome. Additionally, considering the growing evidence on the role of gut-derived immune cells in neuroprotection (5), we proposed that alterations in the oral microbiome could not only impact gut health but also influence the gut-brain axis.

### 1.1 Definition of microbiome

The human microbiome is a complex ecosystem comprising over 10^12^ microorganisms, including bacteria, fungi, archaea, and protozoa, residing in diverse anatomical sites throughout the body, such as the skin, mouth, and gastrointestinal tract (6). Each of these microbial communities has adapted to its specific environment, with unique physical and chemical properties. The gut and oral microbiomes, in particular, are two of the most diverse and populated microbial ecosystems in the human body, playing critical roles in health and disease. These microbiomes play a crucial role in facilitating vital physiological processes, such as digestion, nutrient absorption, immune system modulation, and even mood and cognitive functions (7). The oral microbiome represents one of the most intricate and dynamic assemblages of microbial communities. It contains distinct habitats, such as dental plaque, the tongue, and saliva, which host hundreds of microbial species, including bacteria from phyla like Bacteroidetes, Firmicutes, and Actinobacteria. Additionally, age, diet, and lifestyle factors influence the diversity and composition of the oral microbiome (8). The gut microbiome is similarly complex, with most microbes residing in the large intestine, where they contribute to processes such as the fermentation of non-digestible carbohydrates and synthesis of short-chain fatty acids (SCFAs) (9, 10).

### 1.2 Gut microbiome and systemic disease

The gut microbiome constitutes a heterogeneous and intricate assemblage of microorganisms residing in the gastrointestinal (GI) tract. Among these, the strictly anaerobic lineages of *Firmicutes* and *Bacteroidetes* predominantly thrive in healthy individuals (11). This microbial community is essential for sustaining human health, as it facilitates digestion and modulates the immune system, synthesizing essential nutrients, and protecting against harmful pathogens (11). It facilitates the processes of digestion and nutrient absorption, regulates metabolic functions, modulates the immune system, and provides protection pathogens. This microbial community achieves a delicate balance with its host, promoting overall health (12). However, disruptions to this balance, or dysbiosis, have been implicated in a wide range of systemic diseases, from GI disorders (13) to metabolic (14) and neurodegenerative diseases (15). Gut dysbiosis, characterized by an imbalance in the composition and functionality of the gut microbiome, and has been linked to a variety of diseases (16). For instance, obesity and metabolic syndrome are associated with elevated levels of specific bacterial species that enhance energy extraction from dietary intake, thereby contributing to weight gain (14). Colorectal cancer (CRC) has been associated with an elevation in pro-inflammatory bacterial populations, which can stimulate tumor formation through inflammatory processes and DNA damage (17). Additionally, inflammatory bowel diseases (IBD), which encompass Crohn’s disease and ulcerative colitis, have been correlated with reduced microbial diversity and increased pathogenic bacteria, leading to chronic inflammation and compromised gut barrier function (18).

### 1.3 Gut-brain axis

Emerging research suggests a relationship between the gut microbiome and the central nervous system (CNS), referred to as the gut-brain axis (19). This bidirectional communication pathway involves the nervous system, immune responses, and microbial metabolites. Dysbiosis in the gut has been shown to affect neurological health by compromising the integrity of the blood–brain barrier, resulting in increased permeability and persistent neuro-inflammation. Metabolites, including SCFAs synthesized by gut microbiota, may influence cognitive function and could play a role in the pathogenesis of neurodegenerative disorders such as Alzheimer’s and Parkinson’s disease (20). Recent findings further suggest that gut-derived immune cells, particularly IgA-secreting plasma cells, contribute to CNS immune defense by localizing to the meninges and forming a protective barrier near dural venous sinuses. This highlights the intricate link between gut immunity and brain homeostasis, reinforcing the gut-brain axis as a critical factor in neurological health (5).

### 1.4 Oral microbiome and systemic disease

The oral microbiome represents the second most diverse microbial community within the human body, comprising numerous species of bacteria, fungi, viruses, and archaea. This community inhabits multiple oral environments, such as the teeth, gums, tongue, and saliva, thereby constituting one of the most intricate and dynamic microbial ecosystems in the body, which plays a crucial role in maintaining oral health (21). The composition of the oral microbiome is affected by various factors, including dietary habits and lifestyle, stress, tobacco use and underlying health conditions (22). Disruption of balance within the microbial community can result in dysbiosis, which is linked to oral health issues, including periodontitis (23) and dental caries (24), and has also been correlated with a range of systemic diseases (23).

Dysbiosis of the oral microbiome has been associated with various chronic diseases. One notable example is periodontitis, which is characterized as an inflammatory disorder that impacts the tissues surrounding the teeth, and has been associated with systemic conditions like diabetes, rheumatoid arthritis (RA), cardiovascular diseases, respiratory diseases, proximal colorectal neoplasm, sporadic colorectal cancer, osteoporosis and unfavorable pregnancy outcomes (25–27). Patients with diabetes often experience an overgrowth of bacteria including *Capnocytophaga, Porphyromonas* and *Pseudomonas*, which exacerbate periodontal inflammation (28). In people with RA and systemic lupus erythematosus, elevated levels of bacteria such as *Prevotella* and *Selenomonas* have been observed (29, 30), possibly due to the influence of inflammatory cytokines like interleukin-17 (IL-17) (31). Targeting these cytokines with anti-inflammatory medications may help in restoring microbial balance within the oral cavity, especially in conditions like RA (32).

### 1.5 Systemic implications of oral bacteria

The concept of the oral microbiome’s influence on systemic health was first proposed by Willoughby D. Miller in 1891 (33). Today, it is widely accepted that oral bacteria can impact other body systems (34–36). For instance, certain pathogens from the oral cavity can migrate to other organs, potentially contributing to diseases such as atherosclerosis (37), pneumonia (38), and cancer (39). Oral bacteria like *Streptococcus* species and *Fusobacterium nucleatum* have been associated with various health conditions such as heart disease and colorectal cancer (40, 41). These bacteria are thought to contribute to systemic inflammation and may influence the development and progression of these diseases through direct bacterial translocation or via inflammatory mediators (41).

Recent advances in sequencing and metagenomics analysis have resulted in better understanding of the microbial composition present in the oral cavity and its implications for systemic diseases (25, 42). Personalized treatments, such as probiotics, vaccines, and microbiome-based therapies, are being explored to prevent or manage microbial imbalances (43). Such therapies aim to reduce reliance on antibiotics, address specific microbial profiles, and create a more balanced microbial environment. These advancements highlight the potential for personalized approaches to managing diseases associated with oral dysbiosis, ultimately improving health outcomes by restoring microbial balance (44). There is a continued need for understanding both gut and oral microbiomes in order to formulate targeted and personalized therapeutic strategies within contemporary healthcare.

### 1.6 Oral–gut microbiome

The interaction between the oral and gut microbiomes is crucial in the onset and progression of numerous systemic diseases, encompassing GI disorders and malignancies (45). Studies show that oral bacteria have the capacity to translocate to the gastrointestinal tract, especially in patients with conditions such as bowel cancer and RA (45). Saliva provides a protective environment for these bacteria, which can travel and survive in the GI system (46). Research has shown that specific bacteria from the mouth, such as *Porphyromonas gingivalis*, when present in the gut leads to significant changes in the gut microbiome (47). This translocation can exacerbate inflammatory conditions, including diabetes and colitis, in animal models (48). In cases of periodontitis, patients are found to ingest large amounts of *P. gingivalis* daily, which subsequently modifies the composition of gut microbiome in animal models (45). These bacterial shifts increase the risk of chronic infections and inflammation. For example, in murine models, oral injuries or periodontitis promote the translocation of oral bacteria into the bloodstream, allowing pathogens like *Fusobacterium*, known to be associated with colorectal cancer, to access tumors through the circulatory system (49). Microbial dysbiosis is observed in both the oral and gut microbiome of patients with chronic liver diseases, including cirrhosis (50). This dysbiosis is associated with increased inflammation, impaired liver function, and a higher likelihood of liver cancer (50). The oral-gut microbiome axis has been associated with other cancers, such as pancreatic cancer, suggesting that migration of oral bacteria to the GI tract may contribute to cancerous developments in other organs (51).

Investigations of *P. gingivalis* in mice has demonstrated its impact on systemic inflammation and metabolic changes. When administered orally, *P. gingivalis* significantly alters the gut microbiome, leading to insulin resistance and metabolic endotoxemia—an inflammatory response triggered by toxins from gut bacteria entering the bloodstream (52, 53). Moreover, *P. gingivalis* has the potential to undermine the integrity of the intestinal barrier, leading to increased gut permeability and promoting systemic inflammation, contributing to metabolic disorders (53).

Comprehending the intricate interactions between the oral and gut microbiomes is crucial for advancing strategies in diagnostics, treatment, and personalized medicine. The presence of specific oral pathogens in the gut has been associated with various diseases, including colorectal cancer, liver diseases, and metabolic syndrome. Further research into these pathways could unlock new therapeutic options for managing these conditions.

### 1.7 Oral-brain axis in neurodegenerative disorders

The oral microbiome plays a critical role in both oral and systemic health by supporting immune function, defending against pathogens, and aiding digestion. Emerging research (54) has highlighted its influence on the oral-brain axis—a bidirectional communication pathway connecting the oral microbiome to the brain via neural, immune, and biochemical mechanisms (55). Recent research indicates that dysbiosis within the oral microbiome may play a role in the development of neurodegenerative disorders, including Alzheimer’s and Parkinson’s diseases (56). For instance, studies have reported that individuals with Alzheimer’s disease tend to exhibit lower oral microbial diversity compared to healthy individuals (57). Additionally, research on adolescents has found a strong correlation between higher *α*-diversity of oral microbes and better cognitive function (58). The oral microbiome can impact brain health through the oral-brain axis via mechanisms such as inflammation, pathogen invasion, and metabolic byproducts. Chronic oral infections, such as periodontitis, can release inflammatory cytokines and neurotoxic compounds, including those produced by *Porphyromonas gingivalis*. These compounds may enter the bloodstream, disrupt the blood–brain barrier, or travel along cranial nerves to the brain, triggering neuro-inflammation (59). The detection of specific factors from *P. gingivalis* in systemic circulation supports the hypothesis that the oral microbiome is implicated in the development of neurodegenerative disorders like Parkinson’s disease. These processes may compromise the blood–brain barrier, promote amyloid-*β* accumulation, and exacerbate neurodegenerative pathways linked to Alzheimer’s disease (59). Additionally, microbial byproducts such as lipopolysaccharides (LPS) can intensify neuronal damage and oxidative stress, further contributing to brain health decline (60). Considering the significant influence of oral microbiota on brain health, maintaining oral hygiene and treating oral microbiome dysbiosis may help reduce systemic inflammation and lower the risk of neurodegenerative conditions. This underscores the profound interconnectedness of oral and brain health. Various methods can address oral dysbiosis, potentially benefiting both oral and brain health by supporting a balanced oral microbiota. One such approach is photobiomodulation therapy.

## 2 Photobiomodulation (PBM) therapy

PBM is a non-invasive light therapy employing non-ionizing light sources such as lasers, LEDs, and broadband light to modulate cellular activity without producing heat (61). During PBM, photons are absorbed by intracellular photoacceptors like cytochrome c oxidase in mitochondria and ion channels triggering downstream cellular responses (62). Photon absorption increases cellular energy (ATP production), activates signaling pathways, and modulates cellular responses, leading to benefits in three primary domains: pain and inflammation management, immune system modulation, and enhancement of wound healing and tissue regeneration (62). Nitric oxide (NO) is also released from cytochrome c oxidase, promoting vasodilation and increased blood flow to affected areas. PBM can increase reactive oxygen species (ROS) under normal conditions, stimulating gene expression, protein synthesis, and cellular proliferation (63). Conversely, under oxidative stress, PBM have been shown to diminish levels of ROS, NO, and nuclear factor kappa B (NF-kB), thereby exerting anti-inflammatory effects. This is achieved through the reduction of prostaglandin E2 (PGE2) synthesis and the downregulation of inflammatory cytokines, including interleukins IL-1ß, IL-6, IL-8, IL-12, and tumor necrosis factor alpha (TNFα) (64). Studies have demonstrated PBM’s capacity to activate M1 and M2 macrophages, modulate macrophage phenotypes, activate endogenous, latent transforming growth factor (TGF)-β1 and promote anti-inflammatory cytokine release, indicating its potential as a therapeutic option in conditions expressing chronic inflammation (65, 66).

### 2.1 Photobiomodulation and the microbiome

Photobiomodulation is a potentially innovative approach for modifying dysregulated microbiomes. Bicknell et al. demonstrated that PBM at wavelengths of 660 and 808 nm had an impact on the gut microbiota in mice. Specifically, infrared light notably enhanced the growth of Allobaculum cells (67). In a related investigation utilizing the same wavelength, Thome Lima et al. suggested that PBM may facilitate wound healing in murine models by either eradicating or suppressing the proliferation of *Pantoea agglomerans* bacteria (68). Moreover, research has shown that PBM can hinder bacterial growth by stimulating the generation of ROS, which inflict damage on bacterial cellular components, such as the membrane and deoxyribonucleic acid (DNA) (69). The ability of PBM to interfere with biofilm formation is particularly crucial, as biofilms are associated with chronic infections and antibiotic resistance (70). Moreover, PBM may affect bacterial resistance mechanisms by potentially reducing the expression of resistance genes (71). However, the impact of PBM on bacterial virulence and its potential dual effects in certain circumstances remain subjects of ongoing research. It is important to note that PBM is completely different from antimicrobial photodynamic therapy (aPDT) which uses a photosensitizer to generate reactive oxygen species for direct bacterial eradication (72).

Recent research into “Photobiomics”—the study of how light affects the microbiome—suggests that PBM can positively impact gut microbiome composition, with visible and near-infrared light therapy showing promise (4). PBM’s anti-inflammatory and redox modulation effects are believed to play a role in rebalancing the gut microbiome, which is essential for managing various diseases. PBM influences the gut microbiome by modulating cytokine levels, metabolic pathways, and microbial communities, presenting new avenues for therapeutic interventions, particularly in metabolic and inflammatory diseases (4, 73). Studies have demonstrated PBM’s ability to alter microbiome composition in animal models. For example, research involving red (660 nm) and infrared (808 nm) PBM in mice showed a significant increase in *Allobaculum* (a bacterium linked to gut health) after the infrared exposure (67). The shift in microbial diversity suggests that PBM can be an adjunct therapy for conditions like obesity, cardiovascular disease, and neurodegenerative disorders (67). In clinical settings, PBM may complement existing microbiome-targeted treatments such as probiotics, prebiotics (43), and fecal transplants (74). Research also indicates that the microbiome can be highly responsive to environmental factors such as diet and circadian rhythm (75), the latter being influenced by exposure to light. By influencing microbial activity and reducing inflammation, PBM has the potential to improve treatment efficacy for conditions like Parkinson’s disease (76) and cardiovascular conditions (77). Additionally, PBM therapy may impact metabolic pathways, which could further benefit health outcomes by stabilizing gut flora, reducing oxidative stress, and enhancing antioxidant levels (67).

### 2.2 Photobiomodulation in gut microbiome (GM)-related disorders

PBM therapy has shown considerable potential in managing GM-related disorders by modulating gut microbiome and reducing gut inflammation. PBM has been linked to an increase in beneficial gut bacteria, including *Akkermansia*, *Faecalibacterium*, and *Roseburia*, which are recognized for their role in promoting gut health. Microbial modulation reduces inflammation, oxidative stress, and helps restore microbial balance, contributing to PBM’s therapeutic benefits in GM-related disorders (73).

PBM has shown efficacy in treating gut-related conditions, such as IBD, by reducing inflammation and supporting gut microbial balance. For instance, exposure to narrow-band UVB light demonstrated protective effects against IBD, possibly by promoting vitamin D production and enhancing microbial diversity (78). PBM (at 904 nm) enhanced the prevalence of beneficial bacterial genera, including *Akkermans*ia*, Faecalibacterium*, and *Roseburia*, while simultaneously diminishing the presence of potentially pathogenic genera (79). When near-infrared light at 808 nm was applied for a duration 12 weeks to the abdomen in healthy mice, PBM was associated with increased beneficial *Allobaculum* bacterial species, suggesting a role for PBM in managing chronic inflammatory conditions of the gut (67).

PBM presents a potentially effective strategy for the modulation of the gut microbiome in the treatment of various GM-related disorders, including neurodegenerative and inflammatory diseases. PBM’s ability to influence microbial diversity, reduce inflammation, and support microbial balance makes it a compelling adjunct therapy in the management of gut and systemic health (73).

### 2.3 PBM and neurological disorders with dysbiosis

PBM demonstrated potential in the management of neurological disorders linked to gut dysbiosis, including Alzheimer’s and Parkinson’s diseases (76). Studies in animal models of Alzheimer’s disease have demonstrated that PBM at wavelengths of 630 nm and 730 nm reduced levels of harmful bacteria like *Helicobacter pylori* and Bacteroidales, while increasing beneficial bacteria such as *Rikenella*. This finding suggests that *Rikenella* may serve a protective function and could represent a potential therapeutic target in the treatment of neurodegenerative disorders (80). Furthermore, PBM has been linked to positive shifts in the Firmicutes-to-Bacteroidetes (F:B) ratio in Parkinson’s disease models, indicating a balanced microbiome composition that may alleviate symptoms (81).

The results of recent literature highlights PBM’s potential in modifying gut microbiome, improving neurological symptoms, and offering a non-invasive therapeutic option for people with Parkinson’s disease. Although further research is needed, these findings provide evidence for the potential efficacy of PBM in treating neurological diseases through gut microbiome modulation.

### 2.4 Key parameters of photobiomodulation in oral microbiome management

In a recent published study on the effect of photobiomodulation on oral microbiome dysbiosis, a comprehensive review was done on the PBM parameters (82). The findings revealed that LEDs have demonstrated specific effectiveness based on their wavelengths; for instance, 425 nm (blue light) has been effective in killing *P. gingivalis* and *E. coli DH5α*, while 525 nm (green light) effectively inhibits the growth of *S. aureus*. Conversely, 625 nm (red light) has shown no effectiveness against these pathogens. In addition to LEDs, diode lasers such as those operating at 66, 810, and 940 nm are commonly used for their antimicrobial effects, with 976 nm also noted for reducing *C. albicans* colonies, although not significantly different from the negative control.

The power output of PBM devices is an important factor, with low-power lasers defined as those operating at less than 250 mW. The energy density used in various studies typically ranges from 5 to 100 J/cm^2^, indicating a wide variability in treatment protocols. Exposure times also differ significantly among studies, with LED treatments ranging from 1 to 8 h, while diode laser applications vary from 5 to 100 s. In terms of treatment protocols, LED-mediated PBM has shown a significant reduction in periodontal pathogens, particularly with blue light (400–500 nm). For diode lasers, wavelengths around 810 nm have been noted to be particularly effective against *C. albicans*, especially with exposure times of around 40 s. The observed effects are attributed to mechanisms such as photo-acceptor activation, free radical generation, and subsequent cellular changes that lead to the death of pathogenic bacteria (82).

Moreover, combining PBM with exogenous photosensitizers presents an opportunity to enhance antimicrobial effects through antimicrobial photodynamic therapy (aPDT). While PBM alone has shown promising results, the potential synergistic effects of combining PBM with aPDT could further improve outcomes in managing oral infections. Overall, these findings indicate that while PBM is a promising treatment for oral microbiome dysbiosis, further research is necessary to standardize treatment protocols for optimal effectiveness (82).

## 3 Challenges and limitations of direct photobiomodulation in the gut

The clinical application of PBM directly to the gut is confronted by significant barriers that limit its clinical potential. One of the primary challenges is the difficulty of delivering light to deep tissues within the GI tract. Unlike superficial tissues where PBM can be applied transcutaneously, the gut is surrounded by multiple layers of tissue that act as barriers to light penetration. The gut’s layered structure—with mucosal epithelium, lamina propria, muscularis mucosae, submucosa, muscle layers and serosa— and overlying abdominal tissues significantly attenuate the intensity of incident light (83). Even with optimized near-infrared (NIR) wavelengths (typically in the 760–1,500 nm range, where penetration is maximized), absorption and scattering by these tissues greatly reduce the effective light dose reaching the deeper layers. Beyond light penetration, the optical properties of gastrointestinal tissues further complicate PBM delivery. GI tissues exhibit high variability in scattering coefficients, refractive indices, and absorption characteristics, making it difficult to ensure uniform irradiation of target areas. Chromophores such as hemoglobin, water, and lipids differentially absorb light across the NIR spectrum, leading to heterogeneous energy distribution. Additionally, the high-water content of the gut lining increases absorption in the infrared range, limiting deep tissue effects (84). The gut is not a static organ; rather, it undergoes continuous peristaltic movement, segmental contractions, and changes in luminal volume due to food intake, digestion, and gas production. These dynamic processes introduce several challenges for PBM. The movement of intestinal loops within the abdominal cavity alters the spatial orientation of irradiated tissues over time, leading to inconsistent dosing and affecting reproducibility and therapeutic outcomes (85). Continuous contractions and relaxations of the gut wall may change the distance between the light source and target tissues, leading to fluctuations in energy delivery. In some cases, this movement may reduce effective irradiation time per unit area, diminishing PBM’s biological effects. Additionally, the gut lumen contains a complex mixture of digestive fluids, mucus, and microbiome, all of which affect light transmission and absorption. The presence of partially digested food, bile acids, and intestinal secretions can scatter or absorb light before it reaches target tissues, further complicating dose standardization. Furthermore, the direct application of PBM in GI need invasive procedures, such as endoscopy and colonoscopy, which necessitate anesthesia and incur additional costs. Additionally, since PBM is typically administered over multiple sessions, the direct application does not appear to be a practical approach. While PBM has demonstrated benefits in modulating inflammatory and immune responses, the gut microbiome’s presence and complexity may influence or even counteract these responses in unpredictable ways. Bacterial interactions with host immune cells and gut epithelial cells may alter PBM’s intended outcomes, particularly when dealing with a diverse and shifting microbial community (3). These factors remain to be tested and addressed. It is worth noting that in the future, PDT could offer a more targeted approach by selectively modulating the gut microbiome to achieve desired therapeutic effects. Advances in endoscopic light delivery systems and minimally invasive techniques may also help overcome some of these current limitations. However, further research is needed to refine these strategies and establish clinically viable protocols.

### 3.1 Hypothesis: indirect modulation of gut health via oral microbiome alteration

**Figure 1:**
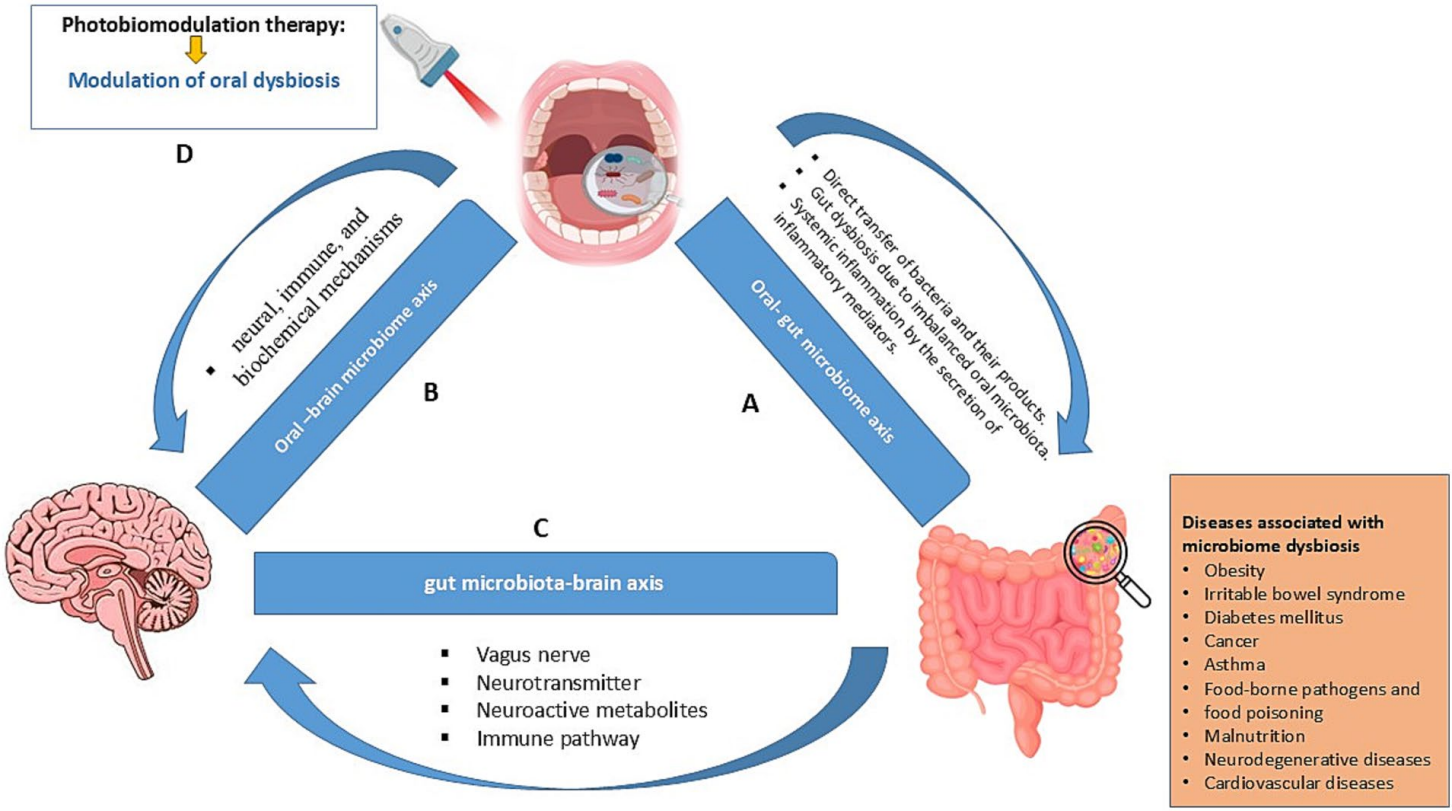
A. The pathway through which oral dysbiosis and infection influence the gut microbiome and their associated disorders; B. The oral microbiome and its interaction pathways with the brain; C. The gut microbiome and its interaction pathways with the brain; D. Hypothesis: PBM could balance the oral microbiome and influence the oral-gut-brain axis.

Considerate of the known and potential limitations, an alternative approach may involve targeting the oral microbiome as an indirect means of influencing gut health. The oral cavity contains a diverse and intricate microbial community that continuously migrates to the gut through swallowing, creating a dynamic relationship between the oral and gut microbiomes (46). Research suggests that changes in the oral microbiome can lead to downstream effects on gut microbial composition, particularly in the upper GI tract where colonization by oral bacteria is common (86). PBM could be applied to the oral cavity to selectively influence the composition and function of the oral microbiome. Previous studies of PBM have shown its capacity to modulate microbial communities by affecting bacterial cell proliferation, biofilm formation, and even specific pathogen suppression (69). By inducing these changes in the oral microbiome, it is conceivable to hypothesis that beneficial alterations could cascade through the GI tract, promoting a healthier composition of gut microbiome and possibly ameliorating gut-related disorders. For instance, an increase in beneficial oral bacteria, such as *Streptococcus salivarius*, which has anti-inflammatory properties, could enhance similar bacterial populations in the gut, contributing to reduced inflammation and improved gut health outcomes (87). Similarly, reducing harmful oral pathogens like *P. gingivalis*, a known contributor to systemic inflammation, could indirectly benefit the gut by lowering overall inflammatory burden (88). This hypothesis, while promising, warrants further research to explore the exact mechanisms and extent of impact that oral PBM-induced changes could have on the gut microbiome. Investigations should aim to identify specific bacterial species affected by PBM in the oral cavity, track their migration, and analyze their influence on gut microbial diversity and host health outcomes. Furthermore, as there may be a direct relationship between oral microbiome and systemic diseases, such as those involving the oral-brain axis, future research may benefit from the application of direct PBM on oral microbiome to manage certain systemic diseases (Figure 1).

## 4 Conclusion and future directions

This article highlights the potential for using PBM to influence human microbial reservoirs. The greatest beneficial effects may be possible at the largest microbial reservoir—the gut. However, direct PBM to the gut has limitations. We introduce an alternative approach: modulating the second largest reservoir of microbes at the oral microbiome to influence gut health. The proposed hypothesis suggests that PBM therapy applied to the oral cavity could result in beneficial shifts in gut microbial composition via the oral-gut microbiome axis. Future research should investigate specific microbial changes induced by oral PBM at both the oral cavity and gut, track microbial migration to the gut, and analyze subsequent effects on gut health. This approach could open new avenues for non-invasive microbiome modulation, offering therapeutic benefits for gut-related and systemic diseases.

## References

[1] Lin D Medeiros DM. The microbiome as a major function of the gastrointestinal tract and its implication in micronutrient metabolism and chronic diseases. Nutr Res. (2023) 112:30–45. doi: 10.1016/j.nutres.2023.02.007, PMID: 36965327

[2] Piquer-Esteban S Ruiz-Ruiz S Arnau V Diaz W Moya A. Exploring the universal healthy human gut microbiota around the world. Comput Struct Biotechnol J. (2022) 20:421–33. doi: 10.1016/j.csbj.2021.12.035, PMID: 35035791 PMC8749183

[3] Laakso EL Ewais T. A holistic perspective on how photobiomodulation may influence fatigue, pain, and depression in inflammatory bowel disease: beyond molecular mechanisms. Biomedicines. (2023) 11:1497. doi: 10.3390/biomedicines11051497, PMID: 37239169 PMC10216148

[4] Liebert A Bicknell B Johnstone DM Gordon LC Kiat H Hamblin MR. “Photobiomics”: can light, including photobiomodulation, alter the microbiome? Photobiomodul Photomed Laser Surg. (2019) 37:681–93. doi: 10.1089/photob.2019.4628, PMID: 31596658 PMC6859693

[5] Fitzpatrick Z Frazer G Ferro A Clare S Bouladoux N Ferdinand J. Gut-educated IgA plasma cells defend the meningeal venous sinuses. Nature. (2020) 587:472–6. doi: 10.1038/s41586-020-2886-4, PMID: 33149302 PMC7748383

[6] Sender R Fuchs S Milo R. Revised estimates for the number of human and bacteria cells in the body. PLoS Biol. (2016) 14:e1002533. doi: 10.1371/journal.pbio.1002533, PMID: 27541692 PMC4991899

[7] Wollam AM Worley KC Wortman JR Young SK Zeng Q Aagaard KM. Structure, function and diversity of the healthy human microbiome. Nature (London). (2012) 486:207–14. doi: 10.1038/nature11234, PMID: 22699609 PMC3564958

[8] Willis JR Gabaldón T. The human oral microbiome in health and disease: from sequences to ecosystems. Microorganisms. (2020) 8:308. doi: 10.3390/microorganisms8020308, PMID: 32102216 PMC7074908

[9] Afzaal M Saeed F Shah YA Hussain M Rabail R Socol CT. Human gut microbiota in health and disease: unveiling the relationship. Front Microbiol. (2022) 13:999001. doi: 10.3389/fmicb.2022.999001, PMID: 36225386 PMC9549250

[10] Portincasa P Bonfrate L Vacca M De Angelis M Farella I Lanza E. Gut microbiota and short chain fatty acids: implications in glucose homeostasis. Int J Mol Sci. (2022) 23:1105. doi: 10.3390/ijms23031105, PMID: 35163038 PMC8835596

[11] Khalil M Di Ciaula A Mahdi L Jaber N Di Palo DM Graziani A. Unraveling the role of the human gut microbiome in health and diseases. Microorganisms. (2024) 12:2333. doi: 10.3390/microorganisms12112333, PMID: 39597722 PMC11596745

[12] McCallum G Tropini C. The gut microbiota and its biogeography. Nat Rev Microbiol. (2024) 22:105–18. doi: 10.1038/s41579-023-00969-0, PMID: 37740073

[13] Pandey H Jain D Tang DW Wong SH Lal D. Gut microbiota in pathophysiology, diagnosis, and therapeutics of inflammatory bowel disease. Intest Res. (2024) 22:15. doi: 10.5217/ir.2023.00080 37935653 PMC10850697

[14] Moreno-Indias I Salgado-Somoza A He A Murri M. Emerging roles of the gut microbiota in the pathogenesis of metabolic disorders. Front Endocrinol. (2021):736371. doi: 10.3389/fendo.2021.736371, PMID: 34484129 PMC8414896

[15] Ashique S Mohanto S Ahmed MG Mishra N Garg A Chellappan DK. Gut-brain axis: a cutting-edge approach to target neurological disorders and potential synbiotic application. Heliyon. (2024) 10:e34092. doi: 10.1016/j.heliyon.2024.e34092, PMID: 39071627 PMC11279763

[16] Winter SE Bäumler AJ. Gut dysbiosis: ecological causes and causative effects on human disease. Proc Natl Acad Sci. (2023) 120:e2316579120. doi: 10.1073/pnas.2316579120, PMID: 38048456 PMC10722970

[17] Jahani-Sherafat S Azimirad M Raeisi H Azizmohammad Looha M Tavakkoli S Ahmadi Amoli H. Alterations in the gut microbiota and their metabolites in human intestinal epithelial cells of patients with colorectal cancer. Mol Biol Rep. (2024) 51:265. doi: 10.1007/s11033-024-09273-3, PMID: 38302841

[18] Haneishi Y Furuya Y Hasegawa M Picarelli A Rossi M Miyamoto J. Inflammatory bowel diseases and gut microbiota. Int J Mol Sci. (2023) 24:3817. doi: 10.3390/ijms24043817 36835245 PMC9958622

[19] Pan I Issac PK Rahman MM Guru A Arockiaraj J. Gut-brain axis a key player to control gut dysbiosis in neurological diseases. Mol Neurobiol. (2023) 61:9873–91. doi: 10.1007/s12035-023-03691-3, PMID: 37851313

[20] Loh JS Mak WQ Tan LKS Ng CX Chan HH Yeow SH. Microbiota–gut–brain axis and its therapeutic applications in neurodegenerative diseases. Signal Transduct Target Ther. (2024) 9:37. doi: 10.1038/s41392-024-01743-1, PMID: 38360862 PMC10869798

[21] Santacroce L Passarelli PC Azzolino D Bottalico L Charitos IA Cazzolla AP. Oral microbiota in human health and disease: a perspective. Exp Biol Med. (2023) 248:1288–301. doi: 10.1177/15353702231187645, PMID: 37688509 PMC10625343

[22] Li X Liu Y Yang X Li C Song Z. The oral microbiota: community composition, influencing factors, pathogenesis, and interventions. Front Microbiol. (2022) 13:895537. doi: 10.3389/fmicb.2022.895537, PMID: 35572634 PMC9100676

[23] Scannapieco FA Dongari-Bagtzoglou A. Dysbiosis revisited: understanding the role of the oral microbiome in the pathogenesis of gingivitis and periodontitis: a critical assessment. J Periodontol. (2021) 92:1071–8. doi: 10.1002/JPER.21-0120, PMID: 33902163 PMC8380683

[24] Zhang JS Chu C-H Yu OY. Oral microbiome and dental caries development. Dentist J. (2022) 10:184. doi: 10.3390/dj10100184, PMID: 36285994 PMC9601200

[25] Peng X Cheng L You Y Tang C Ren B Li Y. Oral microbiota in human systematic diseases. Int J Oral Sci. (2022) 14:14. doi: 10.1038/s41368-022-00163-7, PMID: 35236828 PMC8891310

[26] Kim J Amar S. Periodontal disease and systemic conditions: a bidirectional relationship. Odontology. (2006) 94:10–21. doi: 10.1007/s10266-006-0060-6, PMID: 16998613 PMC2443711

[27] Idrissi Janati A Karp I Latulippe J-F Charlebois P Emami E. Periodontal disease as a risk factor for sporadic colorectal cancer: results from COLDENT study. Cancer Causes Control. (2022) 33:463–72. doi: 10.1007/s10552-021-01541-y 35079924 PMC8821510

[28] Păunică I Giurgiu M Dumitriu AS Păunică S Pantea Stoian AM Martu M-A. The bidirectional relationship between periodontal disease and diabetes mellitus—a review. Diagnostics. (2023) 13:681. doi: 10.3390/diagnostics13040681, PMID: 36832168 PMC9954907

[29] Corrêa JD Calderaro DC Ferreira GA Mendonça SM Fernandes GR Xiao E. Subgingival microbiota dysbiosis in systemic lupus erythematosus: association with periodontal status. Microbiome. (2017) 5:34. doi: 10.1186/s40168-017-0252-z, PMID: 28320468 PMC5359961

[30] Graves DT Corrêa JD Silva TA. The Oral microbiota is modified by systemic diseases. J Dent Res. (2019) 98:148–56. doi: 10.1177/0022034518805739, PMID: 30359170 PMC6761737

[31] Samaan SF Taha SI Mahmoud FA Elsaadawy Y Khalil SA Gamal DM. Role of Interleukin-17 in predicting activity of rheumatoid arthritis and systemic lupus erythematosus. Clin Med Insights Arthritis Musculoskeletal Disord. (2024) 17:11795441241276880. doi: 10.1177/11795441241276880 PMC11440548 39351141

[32] Irie K Azuma T Tomofuji T Yamamoto T. Exploring the role of IL-17A in Oral Dysbiosis-associated periodontitis and its correlation with systemic inflammatory disease. Dent J (Basel). (2023) 11:194. doi: 10.3390/dj11080194, PMID: 37623290 PMC10453731

[33] Miller WD. The human mouth as a focus of infection. Lancet. (1891) 138:340–2. doi: 10.1016/S0140-6736(02)01387-9, PMID: 37496192

[34] Varoni EM Rimondini L. Oral microbiome, oral health and systemic health: a multidirectional link. Biomedicines. (2022) 10:186. doi: 10.3390/biomedicines10010186, PMID: 35052865 PMC8774214

[35] Bourgeois D Gonçalves LS Lima-Junior JC Carrouel F. The oral microbiome is a key factor in oral and systemic health. Front Microbiol. (2022) 13:855668. doi: 10.3389/fmicb.2022.855668, PMID: 35237254 PMC8883028

[36] Baker JL Mark Welch JL Kauffman KM McLean JS He X. The oral microbiome: diversity, biogeography and human health. Nat Rev Microbiol. (2024) 22:89–104. doi: 10.1038/s41579-023-00963-6, PMID: 37700024 PMC11084736

[37] Huang X Xie M Lu X Mei F Song W Liu Y. The roles of periodontal Bacteria in atherosclerosis. Int J Mol Sci. (2023) 24:2861. doi: 10.3390/ijms241612861, PMID: 37629042 PMC10454115

[38] Pathak JL Yan Y Zhang Q Wang L Ge L. The role of oral microbiome in respiratory health and diseases. Respir Med. (2021) 185:106475. doi: 10.1016/j.rmed.2021.106475, PMID: 34049183

[39] Irfan M Delgado RZR Frias-Lopez J. The Oral microbiome and Cancer. Front Immunol. (2020) 11:591088. doi: 10.3389/fimmu.2020.591088, PMID: 33193429 PMC7645040

[40] Fan Z Tang P Li C Yang Q Xu Y Su C. Fusobacterium nucleatum and its associated systemic diseases: epidemiologic studies and possible mechanisms. J Oral Microbiol. (2023) 15:2145729. doi: 10.1080/20002297.2022.2145729 36407281 PMC9673791

[41] Koliarakis I Messaritakis I Nikolouzakis TK Hamilos G Souglakos J Tsiaoussis J. Oral Bacteria and intestinal Dysbiosis in colorectal Cancer. Int J Mol Sci. (2019) 20:4146. doi: 10.3390/ijms20174146 31450675 PMC6747549

[42] Xiao X Liu S Deng H Song Y Zhang L Song Z. Advances in the oral microbiota and rapid detection of oral infectious diseases. Front Microbiol. (2023) 14:1121737. doi: 10.3389/fmicb.2023.1121737 36814562 PMC9939651

[43] Ailioaie LM Litscher G. Probiotics, photobiomodulation, and disease management: controversies and challenges. Int J Mol Sci. (2021) 22:4942. doi: 10.3390/ijms22094942 34066560 PMC8124384

[44] Shukla V Singh S Verma S Verma S Rizvi AA Abbas M. Targeting the microbiome to improve human health with the approach of personalized medicine: latest aspects and current updates. Clin Nutr. (2024) 63:813–20. doi: 10.1016/j.clnesp.2024.08.005, PMID: 39178987

[45] Park S-Y Hwang B-O Lim M Ok S-H Lee S-K Chun K-S. Oral–gut microbiome axis in gastrointestinal disease and cancer. Cancer. (2021) 13:2124. doi: 10.3390/cancers13092124, PMID: 33924899 PMC8125773

[46] Tan X Wang Y Gong T. The interplay between oral microbiota, gut microbiota and systematic diseases. J Oral Microbiol. (2023) 15:2213112. doi: 10.1080/20002297.2023.2213112, PMID: 37200866 PMC10187086

[47] Kato T Yamazaki K Nakajima M Date Y Kikuchi J Hase K. Oral administration of *Porphyromonas gingivalis* alters the gut microbiome and serum metabolome. mSphere. (2018) 3:e00460–18. doi: 10.1128/mSphere.00460-18, PMID: 30333180 PMC6193602

[48] Sohn J Li L Zhang L Settem RP Honma K Sharma A. *Porphyromonas gingivalis* indirectly elicits intestinal inflammation by altering the gut microbiota and disrupting epithelial barrier function through IL9-producing CD4(+) T cells. Mol Oral Microbiol. (2022) 37:42–52. doi: 10.1111/omi.12359 34958712 PMC9353576

[49] Mo S Ru H Huang M Cheng L Mo X Yan L. Oral-intestinal microbiota in colorectal Cancer: inflammation and immunosuppression. J Inflamm Res. (2022) 15:747–59. doi: 10.2147/JIR.S344321, PMID: 35153499 PMC8824753

[50] Acharya C Sahingur SE Bajaj JS. Microbiota, cirrhosis, and the emerging oral-gut-liver axis. JCI Insight. (2017) 2:94416. doi: 10.1172/jci.insight.94416, PMID: 28978799 PMC5841881

[51] McKinley KNL Herremans KM Riner AN Vudatha V Freudenberger DC Hughes SJ. Translocation of Oral microbiota into the pancreatic ductal adenocarcinoma tumor microenvironment. Microorganisms. (2023) 11:1466. doi: 10.3390/microorganisms11061466, PMID: 37374966 PMC10305341

[52] Kang N Zhang Y Xue F Duan J Chen F Cai Y. Periodontitis induced by *Porphyromonas gingivalis* drives impaired glucose metabolism in mice. Front Cell Infect Microbiol. (2022) 12:12. doi: 10.3389/fcimb.2022.998600, PMID: 36299624 PMC9588948

[53] Arimatsu K Yamada H Miyazawa H Minagawa T Nakajima M Ryder MI. Oral pathobiont induces systemic inflammation and metabolic changes associated with alteration of gut microbiota. Sci Rep. (2014) 4:4828. doi: 10.1038/srep04828 24797416 PMC4010932

[54] Wu Y-F Lee W-F Salamanca E Yao W-L Su J-N Wang S-Y. Oral microbiota changes in elderly patients, an Indicator of Alzheimer’s disease. Int J Environ Res Public Health. (2021) 18:4211. doi: 10.3390/ijerph18084211, PMID: 33921182 PMC8071516

[55] Bowland GB Weyrich LS. The oral-microbiome-brain axis and neuropsychiatric disorders: an anthropological perspective. Front Psychol. (2022) 13:810008. doi: 10.3389/fpsyt.2022.810008, PMID: 35432038 PMC9005879

[56] Giordano-Kelhoffer B Lorca C March Llanes J Rábano A Del Ser T Serra A. Oral microbiota, its equilibrium and implications in the pathophysiology of human diseases: a systematic review. Biomedicine. (2022) 10:1803. doi: 10.3390/biomedicines10081803, PMID: 36009350 PMC9405223

[57] Liu S Dashper SG Zhao R. Association between oral bacteria and Alzheimer's disease: a systematic review and Meta-analysis. J Alzheimers Dis. (2023) 91:129–50. doi: 10.3233/JAD-220627, PMID: 36404545

[58] Naumova OY Dobrynin PV Khafizova GV Grigorenko EL. The Association of the Oral Microbiota with cognitive functioning in adolescence. Genes. (2024) 15:1263. doi: 10.3390/genes15101263, PMID: 39457387 PMC11507344

[59] Lei S Li J Yu J Li F Pan Y Chen X. *Porphyromonas gingivalis* bacteremia increases the permeability of the blood-brain barrier via the Mfsd2a/Caveolin-1 mediated transcytosis pathway. Int J Oral Sci. (2023) 15:3. doi: 10.1038/s41368-022-00215-y, PMID: 36631446 PMC9834243

[60] Kalyan M Tousif AH Sonali S Vichitra C Sunanda T Praveenraj SS. Role of endogenous lipopolysaccharides in neurological disorders. Cells. (2022) 11:4038. doi: 10.3390/cells11244038, PMID: 36552802 PMC9777235

[61] De Freitas LF Hamblin MR. Proposed mechanisms of photobiomodulation or low-level light therapy. IEEE J Selected Topics Quant Elect. (2016) 22:348–64. doi: 10.1109/JSTQE.2016.2561201, PMID: 28070154 PMC5215870

[62] Dompe C Moncrieff L Matys J Grzech-Leśniak K Kocherova I Bryja A. Photobiomodulation—underlying mechanism and clinical applications. J Clin Med. (2020) 9:1724. doi: 10.3390/jcm9061724, PMID: 32503238 PMC7356229

[63] Hamblin MR. Mechanisms and mitochondrial redox signaling in Photobiomodulation. Photochem Photobiol. (2018) 94:199–212. doi: 10.1111/php.12864, PMID: 29164625 PMC5844808

[64] Hamblin MR. Mechanisms and applications of the anti-inflammatory effects of photobiomodulation. AIMS Biophys. (2017) 4:337–61. doi: 10.3934/biophy.2017.3.337, PMID: 28748217 PMC5523874

[65] Souza NHC Mesquita-Ferrari RA Rodrigues M da Silva DFT Ribeiro BG Alves AN. Photobiomodulation and different macrophages phenotypes during muscle tissue repair. J Cell Mol Med. (2018) 22:4922–34. doi: 10.1111/jcmm.13757, PMID: 30024093 PMC6156453

[66] Arany PR. Photobiomodulation-activated latent transforming growth factor-β1: a critical clinical therapeutic pathway and an endogenous Optogenetic tool for discovery. Photobiomodul Photomed Laser Surg. (2022) 40:136–47. doi: 10.1089/photob.2021.0109, PMID: 34905400

[67] Bicknell B Liebert A Johnstone D Kiat H. Photobiomodulation of the microbiome: implications for metabolic and inflammatory diseases. Lasers Med Sci. (2019) 34:317–27. doi: 10.1007/s10103-018-2594-6, PMID: 30074108

[68] Thome Lima AMC da Silva Sergio LP da Silva Neto Trajano LA de Souza BP da Motta Mendes JP AFR C. Photobiomodulation by dual-wavelength low-power laser effects on infected pressure ulcers. Lasers Med Sci. (2020) 35:651–60. doi: 10.1007/s10103-019-02862-w, PMID: 31473868

[69] Amaroli A Ravera S Zekiy A Benedicenti S Pasquale C. A narrative review on Oral and periodontal Bacteria microbiota Photobiomodulation, through visible and near-infrared light: from the origins to modern therapies. Int J Mol Sci. (2022) 23:1372. doi: 10.3390/ijms23031372, PMID: 35163296 PMC8836253

[70] Srinivasan R Santhakumari S Poonguzhali P Geetha M Dyavaiah M Xiangmin L. Bacterial biofilm inhibition: a focused review on recent therapeutic strategies for combating the biofilm mediated infections. Front Microbiol. (2021) 12:676458. doi: 10.3389/fmicb.2021.676458, PMID: 34054785 PMC8149761

[71] Kauser A Parisini E Suarato G Castagna R. Light-based anti-biofilm and antibacterial strategies. Pharmaceutics. (2023) 15:2106. doi: 10.3390/pharmaceutics15082106, PMID: 37631320 PMC10457815

[72] Jiang J Lv X Cheng H Yang D Xu W Hu Y. Type I photodynamic antimicrobial therapy: principles, progress, and future perspectives. Acta Biomater. (2024) 177:1–19. doi: 10.1016/j.actbio.2024.02.005, PMID: 38336269

[73] Jahani-Sherafat S Taghavi H Asri N Rezaei Tavirani M Razzaghi Z Rostami-Nejad M. The effectiveness of photobiomodulation therapy in modulation the gut microbiome dysbiosis related diseases. Gastroenterol Hepatol Bed Bench. (2023) 16:386–93. doi: 10.22037/ghfbb.v16i4.2687, PMID: 38313351 PMC10835098

[74] Sahle Z Engidaye G Shenkute Gebreyes D Adenew B Abebe TA. Fecal microbiota transplantation and next-generation therapies: a review on targeting dysbiosis in metabolic disorders and beyond. SAGE Open Med. (2024) 12:20503121241257486. doi: 10.1177/20503121241257486, PMID: 38826830 PMC11143861

[75] Lotti S Dinu M Colombini B Amedei A Sofi F. Circadian rhythms, gut microbiota, and diet: possible implications for health. Nutr Metab Cardiovasc Dis. (2023) 33:1490–500. doi: 10.1016/j.numecd.2023.05.009, PMID: 37246076

[76] Nairuz T Sangwoo-Cho LJ-H. Photobiomodulation therapy on brain: pioneering an innovative approach to revolutionize cognitive dynamics. Cells. (2024) 13:966. doi: 10.3390/cells13110966, PMID: 38891098 PMC11171912

[77] Syed SB Ahmet I Chakir K Morrell CH Arany PR Lakatta EG. Photobiomodulation therapy mitigates cardiovascular aging and improves survival. Lasers Surg Med. (2023) 55:278–93. doi: 10.1002/lsm.23644, PMID: 36821717 PMC10084725

[78] Bosman ES Albert AY Lui H Dutz JP Vallance BA. Skin exposure to narrow band ultraviolet (UVB) light modulates the human intestinal microbiome. Front Microbiol. (2019) 10:2410. doi: 10.3389/fmicb.2019.02410, PMID: 31708890 PMC6821880

[79] Bicknell B Laakso EL Liebert A Kiat H. Modifying the microbiome as a potential mechanism of photobiomodulation: a case report. Photobiomodul Photomed Laser Surg. (2021) 40:88–97. doi: 10.1089/photob.2021.0057 34962422

[80] Chen Q Wu J Dong X Yin H Shi X Su S. Gut flora-targeted photobiomodulation therapy improves senile dementia in an Aß-induced Alzheimer’s disease animal model. J Photochem Photobiol B Biol. (2021) 216:112152. doi: 10.1016/j.jphotobiol.2021.112152, PMID: 33610085

[81] Liebert A Bicknell B Laakso E-L Heller G Jalilitabaei P Tilley S. Improvements in clinical signs of Parkinson’s disease using photobiomodulation: a prospective proof-of-concept study. BMC Neurol. (2021) 21:1–15. doi: 10.1186/s12883-021-02248-y 34215216 PMC8249215

[82] Pourhajibagher M Gharibpour F Nikparto N Bahrami R Bahador A. The effect of photobiomodulation on oral microbiota dysbiosis: a literature review. Photodiagn Photodyn Ther. (2025) 52:104525. doi: 10.1016/j.pdpdt.2025.104525, PMID: 39956443

[83] Coffey JC Byrnes KG Walsh DJ Cunningham RM. Update on the mesentery: structure, function, and role in disease. Lancet Gastroenterol Hepatol. (2022) 7:96–106. doi: 10.1016/S2468-1253(21)00179-5, PMID: 34822760

[84] Ash C Dubec M Donne K Bashford T. Effect of wavelength and beam width on penetration in light-tissue interaction using computational methods. Lasers Med Sci. (2017) 32:1909–18. doi: 10.1007/s10103-017-2317-4, PMID: 28900751 PMC5653719

[85] Van De Graaff KM. Anatomy and physiology of the gastrointestinal tract. Pediatr Infect Dis J. (1986) 5:11–6. doi: 10.1097/00006454-198601001-00005, PMID: 3945583

[86] Zaura E Nicu EA Krom BP Keijser BJ. Acquiring and maintaining a normal oral microbiome: current perspective. Front Cell Infect Microbiol. (2014) 4:85. doi: 10.3389/fcimb.2014.00085, PMID: 25019064 PMC4071637

[87] Kazor CE Mitchell PM Lee AM Stokes LN Loesche WJ Dewhirst FE. Diversity of bacterial populations on the tongue dorsa of patients with halitosis and healthy patients. J Clin Microbiol. (2003) 41:558–63. doi: 10.1128/JCM.41.2.558-563.2003, PMID: 12574246 PMC149706

[88] Kumar PS. Oral microbiota and systemic disease. Anaerobe. (2013) 24:90–3. doi: 10.1016/j.anaerobe.2013.09.010, PMID: 24128801

